# Leveraging social media for youth engagement in HIV prevention: evaluation of the Y-FIT GH open-call campaign in Ghana

**DOI:** 10.1093/heapro/daag154

**Published:** 2026-10-22

**Authors:** Gloria Aidoo-Frimpong, Senam Aku Biddah, Maame Araba Oduro, Abass Tando Abubakar, Yaa Adutwumwaa Obeng, Dorcas Adom, Winfred Kofi Mensah, Brian Ankrah, Daniel Selase Anyidoho, Paul Sowah, Ernest Ortsin, Stephen Ayisi-Addo, Caroline Kingori, Juliet Iwelunmor

**Affiliations:** Department of Epidemiology and Environmental Health, School of Public Health and Professions, University at Buffalo, State University of New York, Buffalo, NY, United States; Center for Interdisciplinary Research on AIDS, Yale University, New Haven, CT, United States; The Thrive Lab at the University at Buffalo, School of Public Health and Health Professions, University at Buffalo, New York, NY, United States; The Thrive Lab at the University at Buffalo, School of Public Health and Health Professions, University at Buffalo, New York, NY, United States; Department of Communication, Ensign College, Salt Lake City, UT, United States; The Thrive Lab at the University at Buffalo, School of Public Health and Health Professions, University at Buffalo, New York, NY, United States; The Thrive Lab at the University at Buffalo, School of Public Health and Health Professions, University at Buffalo, New York, NY, United States; Department of Medical Statistics, Hawa Memorial Saviour Hospital, Osiem Akim-Abuakwa, Ghana; The Thrive Lab at the University at Buffalo, School of Public Health and Health Professions, University at Buffalo, New York, NY, United States; Department of Health Information, St Patrick’s Catholics Hospital, Offinso, Ghana; The Thrive Lab at the University at Buffalo, School of Public Health and Health Professions, University at Buffalo, New York, NY, United States; Department of Behavioral and Community Health Sciences and Policy, Louisiana State University Health Sciences Center, School of Public Health, New Orleans, LA, United States; The Thrive Lab at the University at Buffalo, School of Public Health and Health Professions, University at Buffalo, New York, NY, United States; Department of Laboratory Services, Hawa Memorial Saviour Hospital, Osiem Akim-Abuakwa, Ghana; The Thrive Lab at the University at Buffalo, School of Public Health and Health Professions, University at Buffalo, New York, NY, United States; Department of Health Information, St Michael’s Catholic Hospital, Pramso, Ghana; The Thrive Lab at the University at Buffalo, School of Public Health and Health Professions, University at Buffalo, New York, NY, United States; Department of Health Services, Brigham Young University, Provo, UT, United States; West Africa AIDS Foundation (WAAF), Accra, Ghana; Ghana HIV & AIDS Network, Accra, Ghana; Ghana Health Service, Accra, Ghana; Office of the Provost, Hampton University, Hampton, VA, United States; Division of Infectious Diseases, School of Medicine, Washington University in St. Louis, St Louis, MO, United States

**Keywords:** social media, AYA engagement, HIV self-testing, pre-exposure prophylaxis, long-acting injectable PrEP, health promotion, Ghana, low- and middle-income countries

## Abstract

Adolescents and young adults (AYA) in Ghana continue to experience barriers to HIV prevention, including stigma, limited awareness of HIV self-testing and pre-exposure prophylaxis (PrEP), and insufficient access to AYA-friendly prevention services. This descriptive process evaluation examined how a 2-month, multi-platform communication strategy supported participation in the Youth-Friendly Innovation Tools for HIV Prevention in Ghana national open call for creative HIV self-testing and oral and long-acting PrEP advocacy ideas. The strategy combined paid Facebook advertising, LinkedIn dissemination, and peer-to-peer WhatsApp sharing. Data included platform analytics, dissemination records, application data, and participant-reported awareness sources. Facebook recorded 55 437 impressions and an estimated reach of 48 596 users; LinkedIn generated 650 impressions, 209 engagements, and 171 link clicks. WhatsApp was the dominant self-reported awareness pathway, although individual-level WhatsApp reach, engagement, and conversion could not be measured. A total of 103 eligible AYA submitted written, visual, and audio–visual entries. The findings indicate that organizers can combine broad-reach promotion with peer and institutional networks, mobile-first materials, multiple submission formats, and prospective source tracking. Because exposures overlapped and channel metrics were not equivalent, the study does not establish causal platform superiority. Multi-channel communication can support participatory HIV prevention while requiring offline and assisted options for AYA with limited digital access.

Contribution to Health Promotion

- This study documents how inviting adolescents and young adults (AYA) to create messages, artwork, and videos enabled participatory contributions to HIV prevention communication.
- It describes how public social media, institutional networks, and private peer-sharing channels were combined within one AYA-focused campaign.
- It identifies WhatsApp as the dominant self-reported awareness pathway while acknowledging that individual-level WhatsApp engagement and conversion were not measured.
- The findings provide practical considerations for designing and evaluating AYA-centered digital open calls in Ghana and other resource-constrained settings.

## Introduction

Adolescents and young adults (AYA) aged 15–24 constitute a large population in Ghana. The 2021 Population and Housing Census indicates that the 15–19 and 20–24 age groups together represented approximately one-fifth of Ghana’s 30.8 million residents, about 6.3 million people (Ghana Statistical Service 2021). In 2024, an estimated 37 283 people aged 15–24 were living with HIV, and 4732 new HIV infections occurred in this age group (Ghana AIDS Commission 2024). These estimates underscore the importance of prevention communication that reaches AYA at scale. AYA in Ghana nevertheless face stigma, limited awareness of prevention options, confidentiality concerns, and insufficient access to AYA-friendly services (Ganle *et al*. 2012, Adamu *et al*. 2024, Nartey *et al*. 2025).

HIV self-testing (HIVST) is a process in which a person collects their own specimen, performs an HIV test, and interprets the result; a reactive result requires confirmatory testing according to the national testing algorithm (World Health Organization 2024). HIVST and pre-exposure prophylaxis (PrEP), including oral and long-acting formulations, can expand HIV prevention options for AYA. However, both are largely self-initiated and depend on risk recognition, knowledge, and service-seeking (Hodges-Mameletzis *et al*. 2019). In Ghana, only about one in three AYA report ever testing for HIV (Yu *et al*. 2025), some studies report PrEP awareness near 10% (Owusu *et al*. 2024), and analysis of the 2022 Ghana Demographic and Health Survey found that only 2.4% of women had ever used an HIV self-test kit (Akweh *et al*. 2025). These findings show gaps in knowledge and use, although the estimates arise from different populations and should not be treated as directly comparable national AYA indicators.

HIV communication in Ghana includes facility- and community-based education, school and AYA outreach, peer communication, community organizations, mass media, and increasingly social and mobile media (Adamu *et al*. 2024, Aidoo-Frimpong *et al*. 2024, Tetteh and Kankam 2024). The existence of multiple channels does not guarantee sustained attention or action. Repeated, generalized, or fear-based HIV messaging may contribute to information fatigue and disengagement, as observed among audiences in other African settings (Walker 2022); however, the present study did not directly measure message fatigue. Digital platforms may extend communication through formats already used in everyday interaction, but their functions must be assessed using platform-specific data rather than assumed from platform type. Prior research has shown that social media can support HIV testing, sexual-health education, and recruitment (de Gruchy *et al*. 2021, Stafylis *et al*. 2022, Aidoo-Frimpong *et al*. 2024). Less is known about how paid public promotion, institutional dissemination, and private peer sharing operate together within one time-limited participatory HIV prevention initiative in Ghana.

The Youth-Friendly Innovation Tools for HIV Prevention in Ghana (Y-FIT GH) study addresses this gap by positioning AYA as contributors to prevention innovation (Aidoo-Frimpong *et al.* 2026a, 2026b). Crowdsourcing is an approach in which a large and diverse group is invited to contribute ideas, solutions, or materials to a defined task, after which contributions may be evaluated, shared, or implemented (World Health Organization 2018). Y-FIT GH uses a participatory social innovation model that begins with a national crowdsourcing open call, followed by an innovation sprint and bootcamp to refine selected ideas (Pue et al., 2015). The open call invited AYA across Ghana to submit creative ideas for promoting HIVST and PrEP. It therefore functioned as both a recruitment mechanism and an opportunity for participatory contribution; it did not itself deliver or evaluate HIVST or PrEP services.

Given the national scope and time-bound nature of the open call, the campaign combined paid Facebook advertising, LinkedIn dissemination through institutional and personal accounts, and WhatsApp circulation through statuses, groups, peers, schools, and community organizations. This study describes the campaign’s implementation and examines platform-specific reach, engagement, dissemination records, participant-reported awareness pathways, and eligible applications. It asks how the channels contributed to observed communication activity and participation while recognizing that overlapping exposure and nonequivalent metrics preclude causal comparisons of platform effectiveness.

## Materials and methods

### Study design

This study employed a descriptive process evaluation design to assess the implementation and performance of a social media–based health communication campaign supporting recruitment for the Y-FIT GH national open call (Aidoo-Frimpong *et al.* 2026a, 2026b). The evaluation focused on how different digital communication pathways contributed to dissemination, visibility, and participation during the open-call period. The open call represents the first phase of the broader Y-FIT GH study, an AYA-led social innovation initiative focused on HIV prevention among AYA in Ghana.

Ethical approval was obtained from the University at Buffalo Institutional Review Board (IRB ID: STUDY00009385) and the Ghana Health Service Ethics Review Committee (approval no.: GHS-ERC-030/05/25).

### Open-call procedures and recruitment

The Y-FIT GH open call was conducted from May to July 2025 to solicit youth-generated ideas for promoting HIV prevention among AYA in Ghana. Prior to launch, the study team developed the eligibility criteria, contest messaging, acceptable creative submission formats, and a centralized online application portal. Recruitment followed a multi-channel strategy combining paid Facebook advertising, institutional and partner communication through LinkedIn, and community- and peer-based dissemination through WhatsApp networks. Each channel served a complementary function in extending the reach of the open call across social, institutional, and peer networks. The overall process, from open-call preparation and multi-channel dissemination through application receipt, eligibility screening, and descriptive process evaluation, is summarized in Supplementary Figure S1.

### Campaign implementation

Campaign activities combined organic dissemination and paid advertising. Content consisted primarily of static digital posters, short captions, and links directing interested youth to the centralized application portal, where eligibility criteria and submission instructions were provided. Materials were standardized across platforms to maintain consistency in the core campaign message while allowing dissemination through the distinct functions of each platform. The communication strategy was designed to raise awareness of the open call, encourage youth participation, and facilitate onward sharing through existing social and digital networks.

### Organic dissemination

Organic dissemination was conducted through institutional social media accounts, personal networks of the research team and collaborators, partner organizations, and AYA-serving networks. LinkedIn posts were used to support institutional and professional amplification through reposting and secondary sharing. WhatsApp dissemination occurred through peer-to-peer sharing and group-based distribution within AYA networks, schools, community organizations, and informal social groups. WhatsApp status updates were posted daily throughout the campaign period to sustain visibility and repeated exposure to open-call materials. Group-based dissemination encouraged onward sharing within participants’ own social networks. Due to the private and decentralized nature of WhatsApp communication, engagement metrics for this platform were not directly captured.

### Paid advertising

Paid advertisements were implemented on Facebook to extend campaign reach beyond organic networks. Advertisements were targeted to users in Ghana within the eligible age range for the open call. Targeting parameters included geographic location, age, and interest-based indicators available through the Facebook Ads Manager interface. Paid advertisements used the same core visual assets and messaging as organic posts and linked directly to the application website. Paid dissemination was used to support broad visibility of the campaign, while organic and peer-based dissemination strategies facilitated ongoing circulation and engagement within AYA communication networks.

### Data sources

Campaign performance data were obtained from native platform analytics dashboards, including Facebook Ads Manager and LinkedIn Insights. Metrics extracted included impressions, reach, link clicks, post engagements, page follows, and cost per result for paid advertisements. WhatsApp dissemination metrics were documented based on observed status view counts and records of flyer distribution across WhatsApp groups managed by the campaign team. Organic LinkedIn metrics included impressions, reactions, reposts, and link clicks. Application data were obtained from the open-call submission portal. Participant-reported information sources collected through the application process were also reviewed to examine how AYA encountered and learned about the open call across communication channels. Application counts were aggregated across the campaign period and reviewed alongside campaign activity timelines.

### Operational definitions

For this evaluation, crowdsourcing open call referred to a time-limited invitation for a large and diverse group to contribute ideas or creative solutions to a defined health challenge (World Health Organization 2018). Reach represented the number of unique accounts estimated by a platform to have received content; impressions represented the total number of content displays, including repeat displays; engagement included platform-recorded reactions, shares, clicks, or other interactions; link clicks represented recorded selections of the application link; participant-reported awareness source referred to any channel selected by an applicant as a way they learned about the call; and peer-network dissemination referred to interpersonal or group-based circulation through friends, schools, community organizations, and WhatsApp. Potential WhatsApp group reach was calculated from recorded group membership and was not interpreted as verified exposure, unique reach, or engagement.

### Outcomes

Primary outcomes included digital engagement indicators, measured by impressions, reach, link clicks, and engagement metrics across platforms. Additional outcomes included participant-reported pathways of awareness and indicators of peer-based dissemination across communication environments. A secondary outcome was the number of completed open-call applications submitted during the campaign period. Additional secondary indicators included growth in social media page followership and evidence of secondary dissemination, such as reposting behavior on LinkedIn.

### Data analysis

Descriptive statistics summarized campaign performance separately by platform and dissemination strategy. Facebook reach, impressions, clicks, engagement, and cost metrics; LinkedIn impressions, engagement, clicks, reactions, and reposts; WhatsApp status views and dissemination records; participant-reported awareness sources; and eligible application counts were reported using their source-specific denominators. Metrics were not summed across channels because users and exposures could overlap. No cross-platform conversion rate or causal comparison of channel effectiveness was calculated because channel measures were not equivalent and individual-level WhatsApp engagement and attribution were unavailable.

## Results

### Participant characteristics

A total of 103 AYA submitted eligible entries to the Y-FIT GH open call. Participants were aged 15–24 years (mean, 22.49 years). Most were aged 21–24 years (73, 70.9%), followed by 18–20 years (25, 24.3%) and 15–17 years (5, 4.9%). Females comprised 42 (40.8%) participants and males 61 (59.2%). The largest proportions of submissions were from Ashanti (29, 28.2%), Greater Accra (21, 20.4%), and Savanna (17, 16.5%), with additional entries from nine other regions. Table 1 also presents submission formats. The application dataset did not include sufficiently complete measures of socioeconomic status, disability, phone ownership, data access, or digital literacy for subgroup analysis.

**Table 1:** Sociodemographic characteristics of Y-FIT GH open-call participants (*N* = 103).

Characteristic	*n* (%)
Age group (years)	
15–17	5 (4.9)
18–20	25 (24.3)
21–24	73 (70.9)
Gender	
Female	42 (40.8)
Male	61 (59.2)
Region of residence	
Ashanti	29 (28.2)
Greater Accra	21 (20.4)
Savanna	17 (16.5)
Northern	10 (9.7)
Bono East	7 (6.8)
Upper West	5 (4.9)
Eastern	4 (3.9)
Upper East	3 (2.9)
Volta	3 (2.9)
North East	2 (1.9)
Bono	1 (1.0)
Central	1 (1.0)
Submission format	
Written	33 (32.0)
Poster/graphic design	20 (19.4)
Audio–visual	19 (18.5)
Artwork	15 (14.6)
Photograph	9 (8.7)
Mixed media	7 (6.8)

*N* = 103 eligible entries. Percentages are calculated using the total number of eligible entries.

### Facebook dissemination and engagement

During June 2025, the paid Facebook campaign generated 55 437 impressions and an estimated unique reach of 48 596 users across four ad sets (Table 2). These metrics document broad platform exposure but do not show how many reached users reviewed eligibility information or submitted an entry. The website-promotion advertisement generated 31 082 impressions, reached 27 986 users, and produced 325 link clicks at US dollars (USD) 0.02 per click (USD 6.50 total). Engagement-focused advertisements generated 418 recorded post engagements at USD 0.01 per engagement, and page-like campaigns generated 231 follows or likes at USD 0.03–0.04 per result.

**Table 2:** Paid Facebook advertising performance metrics for the Y-FIT GH open call.

Ad objective	Impressions	Reach	Primary outcome	Total results	Cost per result (USD)	Total cost (USD)
Website traffic (application link)	31 082	27 986	Link clicks	325	0.02	5.89
Post engagement	–	–	Post engagements	418	0.01	–
Page likes/follows	–	–	Page likes/follows	231	0.03–0.04	–
Total (all ad sets)	55 437	48 596	–	–	–	–

### Organic platform performance (LinkedIn)

Organic LinkedIn dissemination comprised four open-call posts shared through institutional and personal accounts. The posts generated 650 impressions, 209 engagements, and 171 link clicks; post-level click-through rates ranged from 6.9% to 37.6% (Table 3). The posts also received 19 reactions and 14 reposts. These results document measured institutional and professional circulation. Because users could encounter content elsewhere and no exclusive attribution was available, the data do not establish LinkedIn’s independent contribution to eligible applications.

**Table 3:** Organic LinkedIn performance metrics for open-call–specific posts.

Metric	Value
Number of posts	4
Total impressions	650
Total engagements	209
Total link clicks	171
Click-through rate (range)	6.9%–37.6%
Reactions	19
Reposts	14
Comments	0

### WhatsApp dissemination metrics

WhatsApp dissemination occurred through daily status posts and repeated flyer sharing across group-based networks from 1 May to 30 June 2025. Status posts averaged ∼120 views. Flyers were distributed to ∼60 groups with an average recorded membership of about 70, corresponding to roughly 4200 potential group memberships (Table 4). This figure is not verified unique reach: group membership could overlap, receipt did not demonstrate viewing, and onward sharing could not be traced. WhatsApp’s private, encrypted structure also prevented direct measurement of individual-level group engagement, link clicks, and conversion to application.

**Table 4:** WhatsApp dissemination metrics for the Y-FIT GH open-call campaign (May–June 2025).

	Description	Value
Campaign dissemination period	Daily WhatsApp status posting	1 May–30 June 2025
Average daily WhatsApp status views	Mean number of views per status post	∼120 views per day
Total days of status posting	Continuous daily posting	∼61 days
WhatsApp group dissemination	Number of groups receiving flyers	∼60 groups
Average group size	Estimated members per group	∼70 members
Estimated potential group reach	Group size × number of groups	∼4200 members
Mode of dissemination	Status posts and group flyer sharing	Repeated, peer-encouraged sharing

### Information sources for open-call awareness

Among the 103 AYA who submitted eligible entries, WhatsApp was the most frequently reported awareness source (75.7%), followed by friends (19.4%), community-based or nongovernmental organizations (9.7%), and schools (7.8%; Table 5). Instagram (4.9%), Facebook (3.9%), and TikTok (1.0%) were reported less frequently as direct sources. Applicants could select more than one source; percentages therefore exceed 100%. These results identify WhatsApp and interpersonal networks as the dominant reported recruitment pathways, but they do not establish that WhatsApp independently caused participation or outperformed other channels on comparable metrics.

**Table 5:** Reported information sources for awareness of the Y-FIT GH open call (*N* = 103).

Information source	*n* (%)
WhatsApp	78 (75.7)
Friend	20 (19.4)
Community or NGO	10 (9.7)
School	8 (7.8)
Instagram	5 (4.9)
Facebook	4 (3.9)
TikTok	1 (1.0)

NGO, nongovernmental organization.

### Cross-channel recruitment pattern

The campaign produced substantially more platform exposure indicators than completed eligible entries: Facebook recorded 55 437 impressions and an estimated unique reach of 48,596, whereas 103 eligible applications were received. A cross-platform conversion rate was not calculated because impressions, reach, link clicks, potential WhatsApp group membership, and participant-reported awareness sources have different denominators and may reflect repeated or overlapping exposure. WhatsApp was the dominant self-reported awareness pathway, and friends were the next most frequently reported interpersonal source. Because peer sharing frequently occurred through WhatsApp and applicants could select multiple sources, the data do not support a causal ranking of platform effectiveness; rather, they indicate that WhatsApp and peer networks were central reported recruitment pathways within the broader campaign.

### Types of creative submissions

AYA participants submitted a diverse range of creative materials in response to the open call (Table 6). Written submissions were the most common format, accounting for 32.0% of entries, and included essays, poems, personal narratives, and scripts addressing HIV awareness, stigma, and AYA empowerment. Posters and graphic designs represented 19.4% of submissions and included digital posters and infographics promoting HIVST and PrEP. Audio–visual submissions accounted for 18.5% of entries and included songs, short videos, podcasts, and interactive demonstrations related to HIV prevention. Artwork submissions, including paintings and drawings, comprised 14.6% of entries, while photographs accounted for 8.7%. Mixed-media submissions that combined text with visual elements represented 6.8% of entries. The diversity of submission formats reflected multiple forms of AYA expression and participation within the initiative, demonstrating how AYA engaged creatively with HIV prevention messaging across visual, written, and audio–visual mediums.

**Table 6:** Types of creative materials submitted by youth participants (*N* = 103).

Submission type	Description	Examples	*n* (%)
Written submissions	Essays, poems, personal narratives, scripts	HIV awareness essays, stigma-focused poems, animation scripts	33 (32.0)
Posters and graphic designs	Digital posters, infographics, mixed-media layouts	Myth-busting posters, HIVST and PrEP promotion visuals	20 (19.4)
Audio–visual submissions	Songs, videos, podcasts, interactive media	HIV songs, HIVST demonstration videos, short podcasts	19 (18.5)
Artwork	Paintings and drawings related to health and identity	Symbolic portraits, abstract thematic art	15 (14.6)
Photographs	Portraits and documentation images	HIVST demonstration photos, self-portraits	9 (8.7)
Mixed-media submissions	Combined text-image or text-art entries	Posters with embedded poetry or quotes	7 (6.8)

## Discussion

This descriptive evaluation shows that the Y-FIT GH open call used multiple communication pathways to generate measurable exposure, engagement, and participation. Facebook recorded the largest measured public-platform reach; LinkedIn recorded institutional and professional engagement, link clicks, and reposts; and WhatsApp was the dominant participant-reported awareness source. These findings support describing the channels as complementary components of the campaign. They do not demonstrate that any platform produced trust, normalization, or movement from awareness to application, because exposure overlapped, WhatsApp engagement was not directly measurable, and the study did not experimentally compare channels.

The contrast between high exposure indicators and 103 eligible applications also requires careful interpretation. Impressions and reach reflect opportunities for exposure, whereas completing a creative open-call entry requires substantially more effort. A conversion rate could not be estimated because channel denominators differed and application sources were overlapping and self-reported. The prominence of WhatsApp and friends is consistent with the relevance of peer-mediated circulation, particularly because peer sharing often occurred within WhatsApp. However, trust, social safety, repeated reinforcement, and information normalization were not measured; these remain plausible mechanisms for future study rather than observed effects of this campaign (Aidoo-Frimpong *et al*. 2024, Yin *et al*. 2024, Niederdeppe *et al*. 2025).

Importantly, the Y-FIT GH open call required a different form of participation than many digital HIV prevention campaigns. Most evaluations of social media in HIV prevention focus on promoting service uptake or recruiting participants into research studies (Liu and Hu 2024). In contrast, AYA participating in the Y-FIT GH open call were invited not only to engage with HIV prevention information but also to contribute creatively to HIV prevention advocacy and innovation. This distinction matters because it changes what constitutes meaningful engagement. Within participatory and mobilization-oriented initiatives, engagement extends beyond clicks, impressions, or application counts to include AYA agency, creativity, and peer leadership within prevention efforts (Canas *et al*. 2019). The diversity of submissions generated through the open call further supports this interpretation. AYA engaged through multiple expressive forms, including writing, visual design, artwork, photography, and audio–visual content, suggesting that the communication strategy created multiple entry points for participation. These findings reinforce broader health promotion scholarship emphasizing the importance of positioning AYA not as passive recipients of health information, but as active contributors to the production, interpretation, and circulation of prevention messaging (Park *et al*. 2017).

### Implications for health promotion in low- and middle-income countries and under-resourced settings

The findings from this study have implications for AYA-centered health promotion in low- and middle-income countries (LMICs) and under-resourced settings, where formal health communication infrastructure may be limited, but everyday social and digital networks are often extensive and highly active (Hagg *et al*. 2018, Ferretti *et al*. 2023). Rather than treating digital communication as a simple mechanism for broadcasting information, this study suggests that health promotion efforts should attend to how prevention messages become visible, credible, and actionable within AYA existing communication ecologies. In the Y-FIT GH open call, AYA engagement did not appear to depend on a single platform or exposure point. Instead, information circulated across public-facing platforms, institutional networks, and private peer communication spaces. This pattern suggests that, in resource-constrained contexts, health promotion may be strengthened by working with the communication infrastructures that AYA already use, rather than relying solely on formal institutional channels.

For stigmatized health issues such as HIV prevention, the central challenge is not only whether information reaches AYA but whether it reaches them through channels that make engagement feel credible, socially safe, and actionable. For HIVST and PrEP, awareness alone may be insufficient if information is not trusted, socially acceptable, or reinforced through familiar networks (Jones *et al*. 2020, Johnson *et al*. 2021, King *et al*. 2022, Temelkovska *et al*. 2023). The prominence of WhatsApp in this study suggests that private messaging environments may function as more than dissemination tools. They may operate as relational spaces where information is encountered repeatedly, endorsed by peers, and normalized through everyday interaction (Hara *et al*. 2014). In settings where AYA may fear judgment or stigma, such private and semi-private spaces may reduce the social risk of engaging with HIV prevention information (Wisniewski 2023). This matters for health promotion because the pathway from information to action is both cognitive and social (Yu and Bekerian 2025). AYA may need to see that prevention information is not only available but also safe to discuss, share, and act upon.

For LMICs and under-resourced settings, these findings also point to the value of low-cost, network-based approaches to health promotion. Paid advertising can extend reach quickly, but it may not be sufficient to generate meaningful participation. In contrast, peer sharing, WhatsApp groups, status posts, school networks, community organizations, and AYA-serving partners can extend the life of a campaign beyond its initial launch. These informal and relational channels are often overlooked because they are harder to measure than impressions or clicks. Yet they may be precisely the channels through which health promotion messages gain credibility and staying power. Designing campaigns for redistribution, rather than only for initial dissemination, may therefore be especially important where resources for repeated paid promotion are limited.

The findings also suggest that AYA-centered health promotion should move beyond treating AYA as target audiences. The open call invited AYA to contribute ideas, narratives, images, and creative materials for HIV prevention. This shifted communication from message delivery to participatory knowledge production. In under-resourced settings, this matters because AYA are not only recipients of public health information; they are interpreters, translators, cultural brokers, and peer messengers (Green *et al*. 2005, Handley *et al*. 2009, Banas *et al*. 2016). Creating multiple forms of participation, including writing, visual design, audio–visual materials, and mixed media, can widen who is able to engage and how they engage (Iwelunmor *et al*. 2022). This is especially important where literacy, access to formal leadership opportunities, and comfort with biomedical language may vary across AYA populations.

Finally, this study raises important implications for how digital health promotion is evaluated. Conventional analytics can document exposure on public-facing platforms, but they do not adequately capture peer diffusion, relational endorsement, or message circulation within private digital environments. This creates a measurement problem for health promotion in LMICs, where some of the most meaningful communication may occur in spaces that are not easily visible to researchers. Evaluation approaches should therefore combine platform metrics with dissemination records, participant-reported information pathways, and evidence of AYA participation and creative contribution. Such approaches avoid overstating precision while recognizing that meaningful health promotion impact often begins before service uptake, in the social processes through which prevention options become known, trusted, and actionable.

### Practical considerations for future open calls

For organizers, the findings support several operational considerations. First, broad-reach advertising should be paired with trusted peer, school, community, and institutional networks rather than used as a stand-alone recruitment strategy. Second, campaign materials should be mobile-first, low-data, discreet, and linked to one clear action, while offering multiple submission formats. Third, unique links, source codes, or brief application questions should be planned prospectively to improve channel attribution, with reach, engagement, completed applications, and conversion treated as distinct indicators. Fourth, teams should document offline and peer dissemination while protecting confidentiality in HIV-related communication. Finally, digital promotion should be complemented by offline publicity, assisted entry, paper-based options, or safe shared-device access so that AYA without personal phones, affordable data, reliable electricity, network coverage, digital literacy, or private device access are not systematically excluded. These recommendations are practice implications from a descriptive campaign evaluation and should be tested in future comparative studies.

### Strengths and limitations

This study has several strengths. First, it evaluates a real-world, time-bound AYA mobilization effort implemented at a national scale rather than a simulated or pilot digital campaign. By documenting communication strategies and dissemination processes as they unfolded, the evaluation captures the operational realities of using digital communication environments to support AYA engagement in HIV prevention within an LMIC context. Second, the study integrates multiple forms of evidence, including platform analytics, documented dissemination activities, and participant-reported pathways of awareness. Bringing these data sources together allowed for a more contextually grounded understanding of how information circulated across interconnected communication environments and helped situate platform metrics within AYA lived experiences of receiving, sharing, and encountering prevention information within their networks. Third, the study moves beyond conventional evaluations of digital health campaigns that focus primarily on exposure metrics or individual platform performance. By examining how communication occurred across public-facing platforms, institutional channels, and private peer networks simultaneously, the study provides insight into the relational and socially embedded nature of AYA engagement with HIV prevention information.

Several limitations should also be considered. Platform analytics did not identify unique users across channels, so total exposure could not be quantified. WhatsApp status views were not unique users; potential group membership was not verified exposure; forwarding chains, link clicks, and individual-level engagement were unavailable. Participant-reported awareness sources could overlap and were subject to recall and attribution error. These limitations preclude causal claims or a definitive ranking of channel effectiveness. The 103 eligible entries were also few relative to recorded exposure indicators, although passive exposure and completion of a creative submission represent different levels of action. Finally, the application form captured a limited set of sociodemographic characteristics, preventing analysis by socioeconomic status, disability, phone ownership, affordability of data, digital literacy, or privacy of device access.

The study also relied partially on self-reported information sources, which may be subject to recall bias or may simplify more complex exposure pathways. AYA may have encountered open-call information through multiple interactions before identifying a primary source of awareness. To address this, participants were permitted to select multiple information sources, and self-reported data were interpreted alongside documented dissemination activities and platform analytics. This approach supported a more nuanced understanding of communication pathways while acknowledging the limits of retrospective self-report.

In addition, the evaluation focused on communication processes and immediate participation outcomes rather than downstream behavioral outcomes such as HIVST uptake, PrEP initiation, or longer-term prevention engagement. This reflects the role of the open call as the entry point into a broader AYA-centered innovation process rather than a direct service-delivery intervention. Although the study does not assess long-term HIV prevention outcomes, it provides insight into how communication environments can support AYA mobilization and participation at the early stages of HIV prevention initiatives. Future phases of the Y-FIT GH study will examine how AYA-generated ideas are refined, implemented, and translated into prevention strategies and community-level impact.

Finally, the campaign depended heavily on digital access and may have underrepresented AYA without personal phones, affordable data, reliable electricity, adequate network coverage, digital literacy, or private access to shared devices, including AYA in remote rural communities. The findings therefore describe communication pathways among applicants who were reached and able to respond, not all AYA in Ghana. Future open calls should pair digital promotion with schools, community organizations, health facilities, offline publicity, assisted entry, paper-based submission, or safe shared-device options. The findings are also situated within Ghana’s sociocultural and communication context and should be adapted rather than assumed to be transferable to other settings.

## Conclusion

This study documents how a multi-channel campaign supported participation in the Y-FIT GH open call. Facebook generated broad measured exposure, LinkedIn contributed recorded institutional engagement and redistribution, and WhatsApp and friends were the dominant self-reported awareness pathways. The study does not establish causal platform superiority or demonstrate that trust, normalization, or social reinforcement produced applications. For practice, organizers should combine public promotion with peer and institutional networks, distinguish exposure from engagement and completed applications, build attribution measures prospectively, protect confidentiality, and provide offline or assisted participation options for AYA facing digital exclusion. These findings offer operational guidance for participatory HIV prevention communication while identifying mechanisms and channel effects that require comparative evaluation.

## Supplementary Material

daag154_Supplementary_Data

## Data Availability

The data supporting the findings of this study are not publicly available due to ethical considerations related to AYA participants and the use of private digital communication platforms. De-identified data may be made available from the corresponding author upon reasonable request, subject to institutional approvals.

## Y-FIT GH Study Team

Anthony Affum Awuah, Ucheoma Nwaozuru, Naa Ashiley Vanderpuye, Temitope Ojo, Joseph Tucker, Oliver Ezechi, Chris Guure, Gamji Abu-Baare, Kwasi Torpey, Emma Gyamerah, Helen Appiah-Ampofo, Michael Gborglah, Enoch Nana Yaw Oduro Agyei, David Caleb Gborglah, Emmanuel Osei-Owusu, Robert Mawuko, Esinu Aku Adza, Juvillar Fugar, Perk Pomeyie, LeatherOnCall Gh, Ebenezer Asare, West Africa AIDS Foundation, THRIVE Lab at the University at Buffalo.
