# Supplementary material for: Leveraging social media for youth engagement in HIV prevention: evaluation of the Y-FIT GH open-call campaign in Ghana
Source: Health Promot Int. 2026 Oct 22;41(5):daag154. doi: 10.1093/heapro/daag154 (PMC13687646; doi:10.1093/heapro/daag154)
Supplement: Supplementary file 1 [file daag154_supplementary_data.pdf]

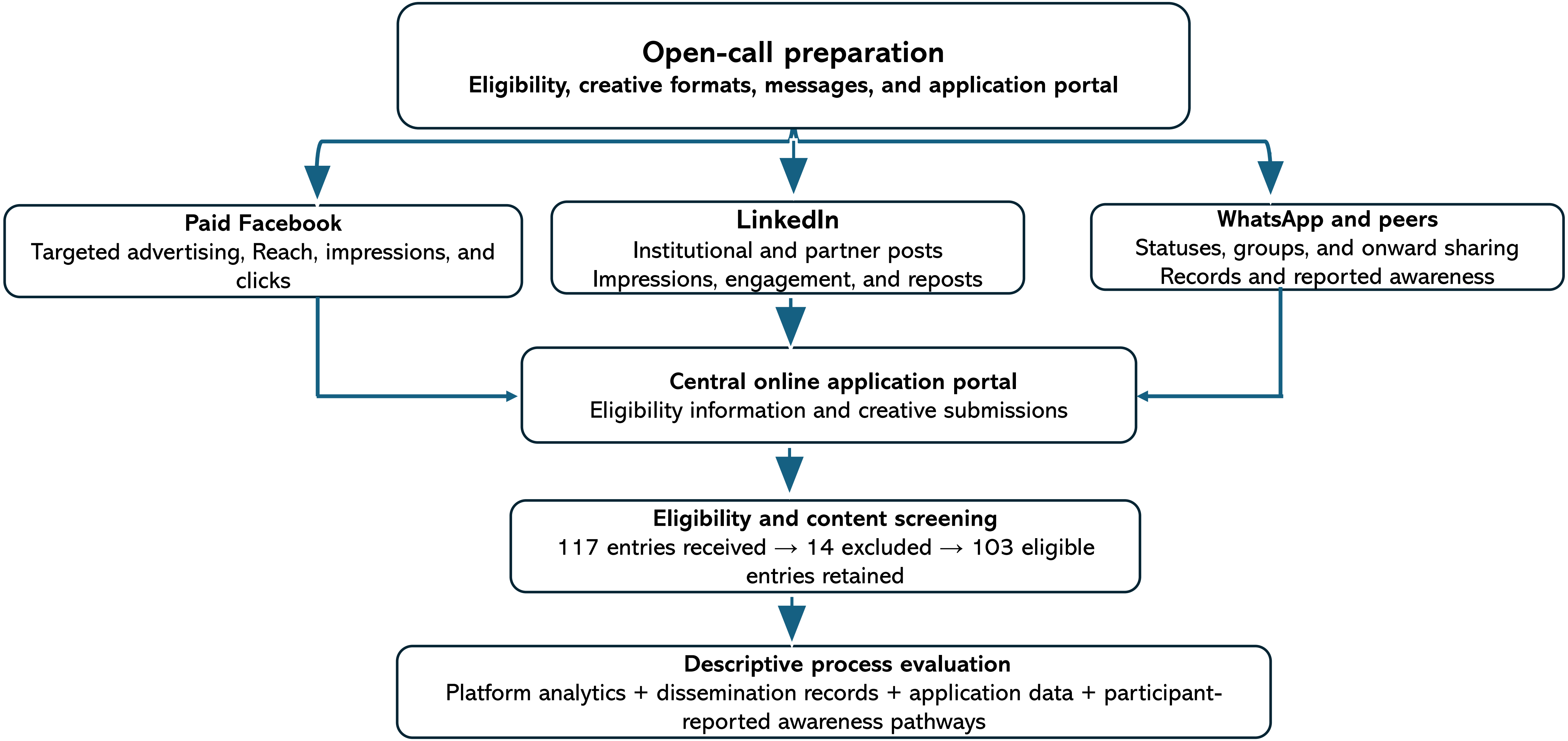

Figure1: Y-FIT GH open-call process and communication pathways
